# Differentially Expressed Nedd4-binding Protein Ndfip1 Protects Neurons Against Methamphetamine-induced Neurotoxicity

**DOI:** 10.1007/s12640-024-00725-z

**Published:** 2025-01-14

**Authors:** Masato Asanuma, Ikuko Miyazaki, Jean Lud Cadet

**Affiliations:** 1 Department of Medical Neurobiology, Okayama University Graduate School of Medicine, Dentistry and Pharmaceutical Sciences, 2-5-1 Shikata-cho, Kita-ku, Okayama, 700-8558 Japan; 2 Molecular Neuropsychiatry Section, Intramural Research Program, NIH/ NIDA, 21224 Baltimore, MD U.S.A.

**Keywords:** Methamphetamine, Neurotoxicity, Nedd4, Ndfip1, Differential display

## Abstract

To identify factors involved in methamphetamine (METH) neurotoxicity, we comprehensively searched for genes which were differentially expressed in mouse striatum after METH administration using differential display (DD) reverse transcription-PCR method and sequent single-strand conformation polymorphism analysis, and found two DD cDNA fragments later identified as mRNA of Nedd4 (neural precursor cell expressed developmentally downregulated 4) WW domain-binding protein 5 (N4WBP5), later named Nedd4 family-interacting protein 1 (Ndfip1). It is an adaptor protein for the binding between Nedd4 of ubiquitin ligase (E3) and target substrate protein for ubiquitination. Northern blot analysis confirmed drastic increases in Ndfip1 mRNA in the striatum after METH injections, and in situ hybridization histochemistry showed that the mRNA expression was increased in the hippocampus and cerebellum at 2 h-2 days, in the cerebral cortex and striatum at 18 h-2 days after single METH administration. The knockdown of Ndfip1 expression with Ndfip1 siRNA significantly aggravated METH-induced neurotoxicity in the cultured monoaminergic neuronal cells. These results suggest that drastic increases in Ndfip1 mRNA is compensatory reaction to protect neurons against METH-induced neurotoxicity.

**Supplementary Information:**

The online version contains supplementary material available at 10.1007/s12640-024-00725-z.

## Introduction

Abused drug methamphetamine (METH) is known to cause neuronal damage to dopaminergic neurons and related neurons. Multiple plausible mechanisms responsible for METH-induced neurotoxicity have been proposed, including reactive oxygen species (ROS), reactive nitrogen species, dopamine (DA) quinone formation, glutamate release, hyperthermia, and proapoptotic molecular events (Ali et al. 1994; Cadet et al. 2003; Jayanthi et al. 2005, 2021; Miyazaki et al. 2006; Shin et al. 2021; Yang et al. 2018), neuroinflammatory processes with microglial activation (Asanuma et al. 2003, 2004; Fantegrossi et al. 2008; Granado et al. 2011; Hozumi et al. 2008; Kays and Yamamoto 2019; Ladenheim et al. 2000; Loftis and Janowsky 2014; Masai et al. 2021; Thomas et al. 2004; Tsuji et al. 2009; Zhang et al. 2006), mitochondrial dysfunction (Brown et al. 2005; Cadet et al. 2005; Dang et al. 2016; Deng et al. 2002; Jayanthi et al. 2001), endoplasmic reticulum (ER) stress (Beauvais et al. 2011; Jayanthi et al. 2009; Krasnova and Cadet 2009; Xiao et al. 2018; Yu et al. 2015), dysfunction of ubiquitin/proteasome system (UPS) (Li et al. 2008; Liao et al. 2005), and autophagic changes (Castino et al. 2008; Huang et al. 2019; Larsen et al. 2002; Li et al. 2017; Lin et al. 2012; Sun et al. 2019; Xu et al. 2018; Yang et al. 2019), predominantly in the striatum. To further identify factors involved in METH neurotoxicity, we comprehensively searched for genes that were differentially expressed after METH administration in the striatum using differential display reverse transcription-PCR (DD RT-PCR) method and sequent single-strand conformation polymorphism (SSCP) analysis, and found two differentially displayed cDNA fragments of expression sequence tags (ESTs) in 1998. Later, the both gene were identified as mRNA of Nedd4 (neural precursor cell expressed developmentally downregulated 4) WW domain-binding protein 5 (N4WBP5), later named Nedd4 family-interacting protein 1 (Ndfip1), which is an adaptor protein for the binding between Nedd4 of ubiquitin ligase (E3) and target substrate protein for ubiquitination (Harvey et al. 2002; Jolliffe et al. 2000). To our knowledge, few studies show involvement of Nedd4 or Ndfip1 on METH neurotoxicity. In the present study, therefore, we have examined expression of Ndfip1 mRNA and its protein in METH-treated animals and also investigate involvement of Ndfip1 in METH-induced neurotoxicity by knockdown of Ndfip1 expression in METH-treated cultured neuronal cells.

## Materials and Methods 

### Reagents and Animals

TRIzol Reagent and Dulbecco’s modified Eagle’s medium (DMEM) were purchased from Invitrogen (ThermoFisher Scientific, San Diego, CA, USA). A differential display kit (Delta differential display kit), RT-PCR kit (Advantage RT-for-PCR kit), 3’-terminal deoxynucleotidyl transferase, Mouse Multiple Tissue Northern Blot were from Clontech (CA, USA). [α-^33^P]dATP, [α-^32^P]dCTP and a T7 Sequenase version 2.0 DNA sequencing kit were purchased from Amersham Life Science (IL, USA). Male CD-1 mice (Charles River, NC, USA), weighing 30–35 g, were used in the present study. All animal use procedures were according to the NIH Guide for the Care and Use of Laboratory Animals, and were approved by the local NIDA Animal Care Committee by the Animal Care.

### Drug Administration and RNA Extraction

For differential display PCR, mice were given METH injections (5 mg/kg, i.p. ×4 with 2 h-interval) and then were killed at 1 day or 14 days after the last injection. Control animals were given 4 injections of saline. The brains were immediately removed and the striatum dissected out on ice. Total RNA was extracted from dissected striatal tissue using TRIzol Reagent. RNA extracts (0.67 µg/µl) from five separate animals in each group were mixed, and purified by incubation with DNase I (Ambion, TX, USA) and phenol/chloroform, chloroform/isoamylalcohol extractions. The concentration of total RNA was measured using a spectrophotometer.

### Differential Display Reverse Transcription RT-PCR (DD RT-PCR)

Arbitrarily primed PCR was performed using the Delta differential display kit (Clontech) according to the manufacturer’s instructions. Briefly, total RNA (1 µg) was mixed with 2 pmol of oligo (dT)18 primers (Clontech), incubated at 70 °C for 3 min, and then placed on ice. The reaction, 5 µl was mixed with 2 µl of 5× reaction buffer, 10 nmol of dNTP, 20U of RNase inhibitor and 200U of Moloney-Murine Leukemia Virus (MMLV) reverse transcriptase (Advantage RT-for-PCR kit; Clontech) in total volume 10 µl, and incubated at 42 °C for 1 h. Final reaction was heated at 75 °C for 10 min and then placed on ice. After dilution of this single stranded cDNA to 1/100, 1 µl of diluted cDNA was mixed with 2 µl of 10× KlenTaq buffer, 1 nmol of dNTP, 0.2 µl of [α-^33^P]dATP (Amersham Life Science), 0.4 µl of 50× KlenTaq polymerase (Clontech), 20 pmol of P8 forward primers (Delta differential display kit; ATTAACCCTCACTAAATGGAGCTGG), and 20 pmol of T8 reverse primers (Delta differential display kit; CATTATGCTGAGTGATATCTTTTTTTTTGC) in a total volume of 20 µl. For negative controls of PCR, 1/100 diluted total RNA or H_2_O was used as the template instead of cDNA. Arbitrarily primed PCR were performed in a Perkin-Elmer DNA Thermal Cycler 480 under following conditions: 1 cycle of 94 °C 5 min, 40 °C 5 min, 68 °C 5 min; 1 cycle of 94 °C 2 min, 40 °C 5 min, 68 °C 5 min; 22 cycles of 94 °C 1 min, 40 °C 1 min, 68 °C 2 min; 68 °C 7 min; 4 °C hold. The PCR reaction (2.5 µl) was mixed with formamide loading dye (95% formamide, 0.2% xylene cyanol, 0.2% bromophenol blue in 12.5 mM EDTA), and then loaded onto a 5% polyacrylamide/8 M urea gel (380 × 500 × 0.4 mm) for 2.5 h at 120 W in 0.5X Tris-borate-EDTA (TBE) buffer. The gel was dried on filter paper, and exposed to Biomax-MR film (Kodak, NY, U.S.A.) at −70 °C for 5 days. Differentially expressed bands in METH-treated group which were absent in total RNA and H_2_O negative controls and cDNA in saline-treated group were excised from the gel, purified by ethanol precipitation, and then resuspended in 80 µl of Tris/EDTA (TE) buffer (pH 7.4).

### Single-strand Conformation Polymorphism (SSCP) Analysis

To separate multiple DNAs in one differentially expressed band, SSCP was performed according to the method previously reported (Murakami et al. 1991) with minor modification. Eluted DNA (2 µl) was reamplified by PCR using the same reagents as the arbitrarily primed PCR under 20 cycles of 94 °C 1 min, 40 °C 1 min, 68 °C 2 min. PCR mixture (5 µl) was heated with 45 µl of formamide loading dye at 80 °C for 5 min, and 2 µl of preparation was then applied to non-denatured 5% polyacrylamide gel (380 × 500 × 0.4 mm) containing 10% glycerol for 24 h at 10 W in 0.5× TBE buffer. The gel was dried and exposed to Biomax-MR film (Kodak) at −70 °C for 5 days. Differential expressed bands in METH-treated group on the SSCP gel were excised, rehydrated in 50 µl of TE buffer. Extract from cut bands was reamplified with the same PCR without [α-^33^P]dATP.

### Direct Sequencing by Asymmetric PCR

To make single stranded DNA template for direct sequencing of differentially expressed SSCP bands (8CB and 8CC), asymmetric PCR was carried using 100-folds reverse primers. The reamplified DNA (5 µl) was mixed with 2 µl of 10× KlenTaq buffer, 1 nmol of dNTP, 0.4 µl of 50× KlenTaq polymerase (Clontech), 0.2 pmol of P8 forward primers, and 20 pmol of T8 reverse primers in a total volume of 20 µl. Asymmetric PCR was performed under 20 cycles of 94 °C 1 min, 40 °C 1 min, 68 °C 2 min. After removal of primers, concentration of asymmetric PCR products was measured with a spectrophotometer. As templates and primers for direct sequencing, 0.54 pmol of asymmetric PCR product which is mostly anti-sense strand and 4 pmol of P8 forward primers were used, respectively. Direct sequencing was performed by using a T7 Sequenase version 2.0 DNA sequencing kit (Amersham) according to the manufacturer’s instructions with dITP. Sequences were compared to the GenBank + EMBL + DDBJ + PDB at the National Library of Medicine, using the blastn algorithm on the non-redundant database at their online website in 1998. Novel sequences were further compared with the non-redundant GenBank mouse expressed sequence tags (ESTs) database. The sequences differentially expressed were again compared to the GenBank in 2001 and 2006.

### Northern Blot Analysis

To confirm the specificity of differential expression in DD RT-PCR/SSCP, Northern blot analysis was performed using the asymmetric PCR product as probes. Total RNA (10 µg/lane) from striatum at 14 days after METH injections (5 mg/kg, i.p. ×4 with 2 h-interval) or saline injection (*n* = 5 mice) was denatured with 6.7% formaldehyde and 50% formamide, electrophoresed on a 1% denaturing agarose/formaldehyde gel and transblotted directly onto nylon membranes (Hybond-N, Amersham). The blotted RNA was fixed by baking. Mouse Multiple Tissue Northern Blot (Clontech) was also used to clarify the distribution of the differentially expressed genes. To make radiorabeled anti-sense probes for differentially expressed 8CB + 8CC mRNA, asymmetric PCR was performed using 0.2 pmol of P8 forward primers, and 20 pmol of T8 reverse primers and [α-^32^P]dCTP (Amersham) under 20 cycles of 94 °C 1 min, 40 °C 1 min, 68 °C 2 min. To make negative control sense probes, asymmetric PCR using 20 pmol of P8 forward primers and 0.2 pmol of T8 reverse primers was also performed. Asymmetric PCR probes were purified by chloroform extraction. As internal control probes, synthetic 40-mer β-actin oligonucleotide probes (New England Nuclear, MA, USA) with a sequence complementary to bases 394–434 of the β-actin mRNA were radiolabeled on the 3’-ends with [^32^P]dCTP by 3’-terminal deoxynucleotidyl transferase using a commercially available kit (Amersham). Blots were prehybridized for 4 h at 42 °C in hybridization buffer, containing 5× SSC (1× SSC; 0.15 M NaCl, 0.015 M sodium citrate, pH 7.0), 5× Denhardt’s solution, 50% formamide, 0.1% SDS, 2.5% dextran sulfate, and 100 µg/ml denatured salmon sperm DNA. Hybridization was performed for 18 h at 42 °C in the same buffer containing approximate 10^6^ dpm/ml ^32^P-labeled probes. After hybridization, membranes were washed in 1× SSC containing 0.1% SDS for 15 min at room temperature, followed by 15 min at 50 °C twice, and exposed to X-ray film (Hyperfilm MP, Amersham) for 10 days at −70 °C. After reprobing, blots were rehybridized with β-actin mRNA oligonucleotide probes as on the initial blots to normalize for loading and transfer artifacts introduced in Northern blotting.

### In Situ Hybridization

For in situ hybridization, mice were given single METH injection (20 mg/kg, i.p.) (*n* = 4 mice/ each time point) and then were killed at 2, 4, 18 h, 1, 2 or 7 days after the injection to examine a spatiotemporal expression of the differentially expressed genes. Control animals were given injection of saline (0 h). The time from decapitation to freezing of the removed brains with powdered dry ice was kept constant. Freshly frozen brain blocks were cut into 20 μm-thick of medial and lateral sagittal sections (1.2 mm and 2.3 mm lateral to the midline, respectively) with a cryostat at −25 °C. Sections were mounted directly onto gelatin-coated slides and air-dried. Mounted sections were fixed in 4% paraformaldehyde/ 0.1 M sodium phosphate buffer (PB; pH 7.4) for 30 min at room temperature, rinsed in 0.1 M PB for 20 min, and then prehybridized for 1 h with a solution containing 1× Denhardt’s solution and 2× SSC. The ^33^P-labeled anti-sense and sense probes were prepared by asymmetric PCR as described in Northern blot analysis section using ^33^P -dATP. The sections were hybridized for 18 h at 42 °C with the [^33^P]-labeled probes (approx. 10^6^ dpm/ml) in the hybridization buffer under Parafilm coverslips in a humid chamber. The hybridization buffer included 10 mM dithiothreitol, 50% formamide, 5× SSC, 5× Denhardt’s solution, 200 µg/ml denatured salmon sperm DNA, and 2.5% dextran sulfate. The sections were washed in 1× SSC for 15 min at room temperature twice, followed by 15 min at 50 °C twice, and then dehydrated with ethanol. Non-specific hybridization (negative control) was determined by incubation with the [^33^P]-labeled sense probes that were synthesized by asymmetric PCR. All washed sections were exposed to X-ray films (Hyperfilm beta-max film, Amersham) at −70 °C for 14 days. Each brain region was identified according to the anatomical atlas.

### Cell Culture, SiRNA Lipofection and METH Treatment

The rat monoaminergic neuroblastoma cell line, B65 neuronal cells (ECACC; #85042305, Salisbury, Wiltshire, UK) were continuously cultured in DMEM (ThermoFisher Scientific) supplemented with 10% FBS, 2 mM L-glutamine and 60 µg/ml kanamycin sulfate. Cells were seeded onto 6-well plates and 96-well plates (Falcon, Corning, NY, USA) at a density of 2.3 × 10^4^ cells/cm^2^ and 3.1 × 10^4^ cells/cm^2^ for western blot analysis and the morphological study with siRNA lipofection, respectively. Cells were maintained in the culture medium at 37ºC in a 5%/95% CO_2_/air mixture.

For western blot analysis on dose-dependency of METH-induced Ndfip1 expression, 2 days after cultivation, B65 cells were treated with METH (final concentration: 500 µM or 1 mM) for 3 h.

For siRNA lipofection assay to knockdown Ndfip1 expression, three 21-mer siRNAs targeted to Ndfip1 mRNA with 3’-overhanged with dTdT (#1 targeted to 510–528 or 459–477, #2 targeted to 785–803 or 734–752, and #3 targeted to 1359–1377 or 1308–1326 of N4WBP5 (Ndfip1) mRNA (accession number: AF220209 or BC020359, respectively)) were synthesized and annealed (B-Bridge International, Inc., CA, USA). Negative control double-stranded RNA (C5A-0600) was also used as a negative control siRNA. Mixed Ndfip1 siRNAs or negative control siRNA were preincubated with liposome (lipofectamine RNAiMAX, ThermoFisher Scientific) for 20 min at room temperature, then the liposomed siRNAs (final concentration of siRNA: 50 nM) were transfected into cultured B65 cells on 6-well plates for western blot analysis or on 96-well plates for morphological studies in serum-free DMEM for 6 h. Next day replacing with serum-containing DMEM, cells were treated with 250 µM, 500 µM or 1 mM of METH for 3, 6, or 24 h, then conducting western blot analysis or morphological studies.

### Western Blot Analysis

For western blot analyses, cytosolic lysates from the cells were extracted using the NE-PER™ Nuclear and Cytoplasmic Extraction Reagents (ThermoFisher Scientific). Cultured neuronal cells were lysed by incubation in ice-cold Cytoplasmic Extraction Reagent with protease inhibitor cocktail (ThermoFisher Scientific) for 10 min. After centrifugation (15,000 g, 5 min at 4 °C), supernatants were collected. Protein concentrations were measured using the Bio-Rad DC protein assay kit (#5000112JA, Bio-Rad, Richmond, CA, USA).

Protein samples were separated on Any kD SDS-polyacrylamide gels (#4569035, Bio-Rad) and electrophoretically transferred to PVDF membranes (Immobilon-P, Millipore, Temecula, CA, USA). Blots were incubated with mouse monoclonal anti-Ndfip1 (1:1,000, sc-515417, Santa Cruz Biotechnology, Santa Cruz, CA, USA) or goat polyclonal anti-β-actin (1:250, sc-1615, Santa Cruz Biotechnology) followed by incubation with the corresponding HRP-conjugated secondary antibody. Chemiluminescent signals were visualized using the ECL western blotting detection system (GE Healthcare UK, Buckinghamshire, UK). Images were quantified using a FUJIFILM Luminescent Image Analyzer LAS-3000 (FUJIFILM, Tokyo, Japan) and Multi Gauge (v3.0) software. For quantitative analysis, the signal intensity ratio Ndfip1/β-actin was calculated to normalize for loading and transblot artifact.

### Morphological Study

Morphological changes of both cells were studied by microscopic observation. Significant morphological changes include disappearance, shrinkage of dendrites, shrunken round-shape cell body, or vacuolation in cytoplasm. All slides stained by Hoechst dye were analyzed under a fluorescence microscope (Olympus BX50-FLA, Tokyo, Japan), using a mercury lamp through a 360–370 nm band-pass filter to excite Hoechst dye. Light emitted from Hoechst was collected through 420 nm long-pass filter. Fluorescence intensity was analyzed using ImageJ software (NIH, Bethesda, MD, USA).

### Statistical Analyses

All statistical analyses were performed using KaleidaGraph v4.0 software (HULINKS Inc., Tokyo, Japan). Data are presented as means ± standard error of the mean (SEM) to show the reliability and accuracy of the mean value. Differences between groups were examined for statistical significance using one-way ANOVA or two-way ANOVA followed by Fisher’s PLSD *post hoc* test. A *p*-value less than 0.05 denoted the presence of a statistically significant difference.

## Results

### DD RT-PCR/SSCP

**Fig. 1:**
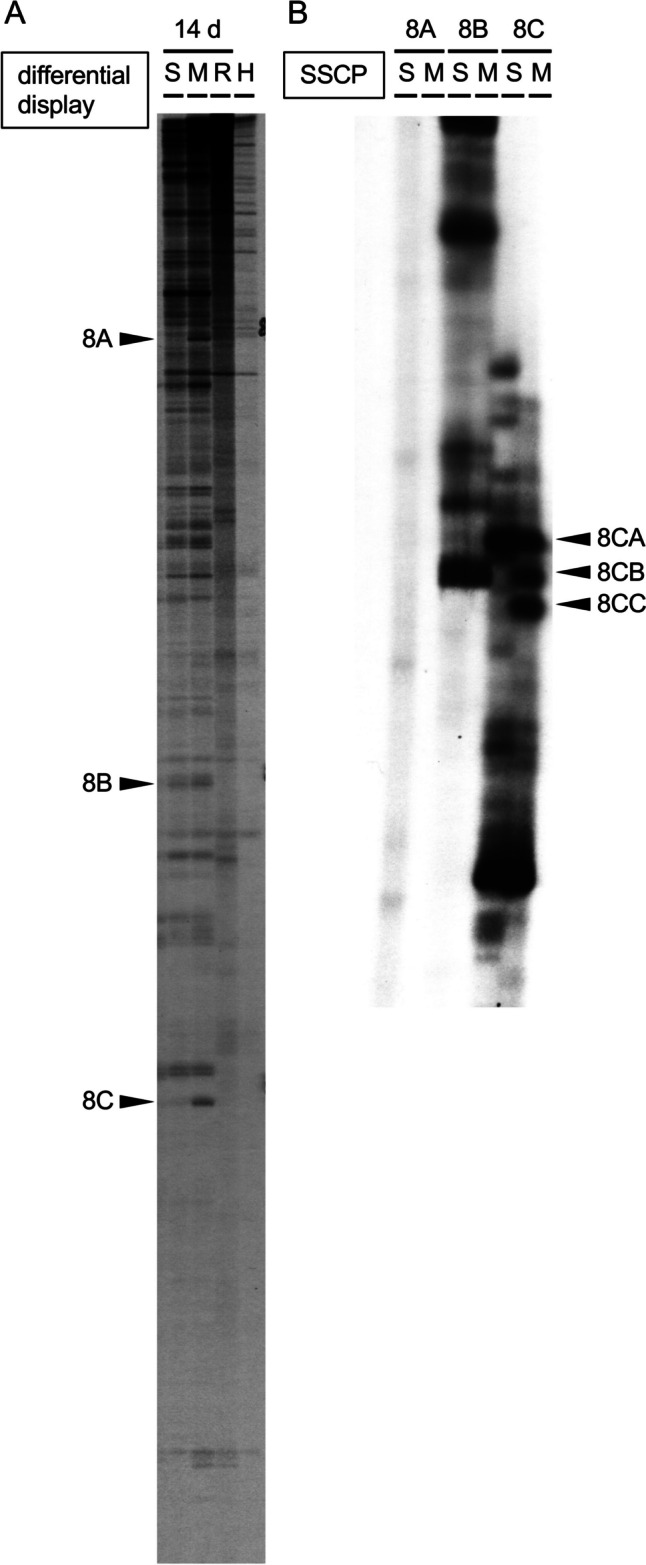
**A**: DD analysis with RT-PCR using cDNA from the striatum 14 days after METH injections (5 mg/kg, i.p. ×4 with 2 h-interval). Equal amount of 0.67 µg/µl striatal total RNA extracts from five animals in each group were mixed, and 1 µg of total RNA extract mixture in each group was used. Differentially expressed bands of 8 A, 8B and 8 C were observed in METH-treated group (M), which were absent in 1/100 diluted total RNA (R) or H_2_O (H) negative controls and cDNA in saline-treated group (S). **B**: SSCP of differentially displayed 8A, 8B and 8C fragments. SSCP separated two differentially expressed bands (8CB and 8CC) from 8C DD product in METH-treated mouse striatum

In DD RT-PCR using a primer set of P8 and T8, we obtained three clearly differentially expressed bands in the striatum at 14 days after METH administration (5 mg/kg, i.p. ×4 with 2 h-interval) (Fig. 1A: bands of 8 A, 8B and 8 C). Although the differentially displayed band looks single, it often includes several different cDNA fragments. To separate multiple cDNAs in one differentially expressed band, we performed SSCP using non-denaturing acrylamide gels. The SSCP showed two actually differentially expressed bands (8CB and 8CC) in 8 C DD product from METH-treated mouse striatum (Fig. 1B: 8CB and 8CC).

### Sequence Analysis of SSCP Bands

We reamplified those differentially expressed SSCP bands (8CB and 8CC), and did direct sequencing by asymmetric PCR to make single anti-sense strand as the sequencing template. The sequences of both gene fragments are quite overlapped each other (Table 1). These 8CB and 8CC fragments have no homology to known complete full-length cDNAs in the GenBank + EMBL + DDBJ + PDB databases in 1998. However, further search of the GenBank mouse EST databases showed significant homologies of both fragments to various EST clones. Especially, both our gene fragments were quite identical (100% homologous) to 12 of mouse ESTs (accession number: AA122516, AA571530, AA175138, AA204138, AA259929, AA501000, AA422537, AA268257, AA209005, AA790336, AA200752, AA711249). Later in 2001 and 2006, the differentially expressed fragments were again compared to the GenBank, and were found to 100% identical to N4WBP5 (later named Ndfip1) mRNA (accession number: AF220209) and Ndfip1 mRNA (accession number: BC026372 or BC020359).

**Table 1:**
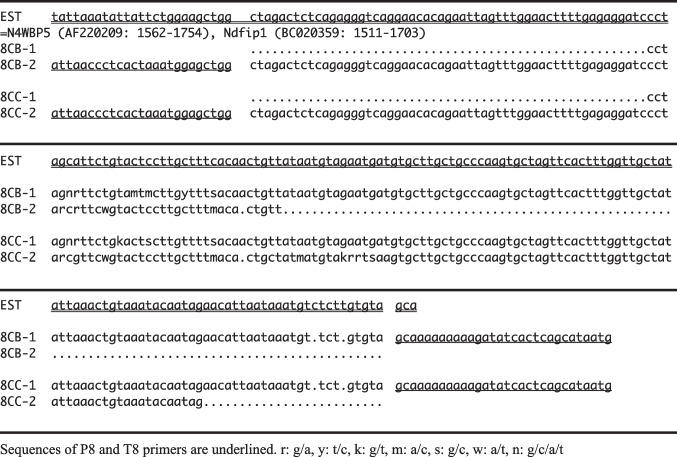
Sequences of differentially expressed SSCP bands (8CB and 8CC) from METH-treated mouse striatum

### Ndfip1 (N4WBP5) MRNA and Its Protein Expression in Mouse

**Fig. 2:**
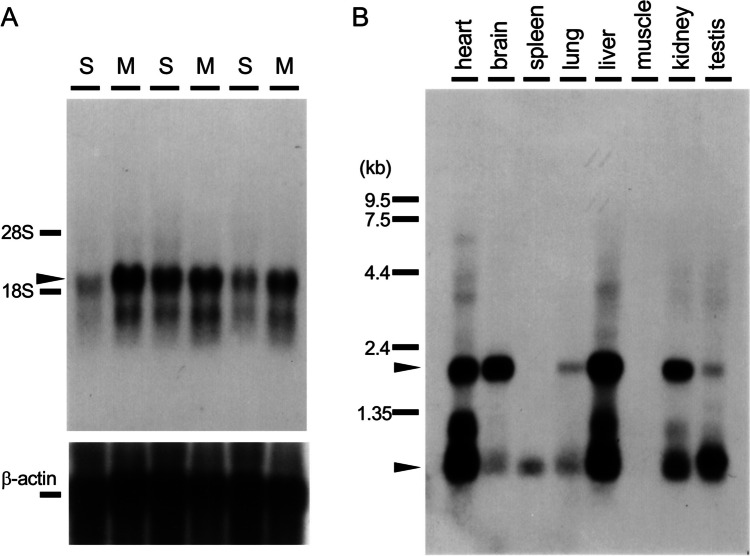
**A**: Northern blot hybridization analysis of differentially expressed 8CB + 8CC gene fragments, which later identified to Ndfip1 (N4WBP5) mRNA in the striatum 14 days after METH injections (5 mg/kg, i.p. ×4 with 2 h-interval) (M: *n* = 5 mice) or saline injections (S: *n* = 5 mice) using ^32^P-labeled asymmetric PCR probes for 8C fragments. Total RNA (10 µg/lane) was loaded. **B**: The expression of 8C fragments (N4WBP5, Ndfip1 mRNA) on Multiple Tissue Northern Blot to clarify the tissue distribution of the genes

To clarify the specificity of differential expression of these gene fragments in METH-treated animals, we examined Northern blot hybridization using anti-sense probes of 8CB + 8CC mRNA, which later identified to Ndfip1 (N4WBP5). Northern blot analysis confirmed drastic increases in the mRNA of these gene fragments in the striatum at 14 days after METH injections (5 mg/kg, i.p. ×4 with 2 h-interval) (Fig. 2A). To study the systemic distribution of the mRNA, we also used a blot which has mRNA from various mouse tissues. The Multiple Tissue Northern Blot showed 2 bands of mRNA (Fig. 2B). The upper long mRNA (approx. 2000 bases) was seen in the brain, liver, heart and kidney. On the other hand, the lower band (approx. 800 bases) was strongly seen in the heart, liver and testis, but it was moderate in the kidney. Interestingly, longer mRNA was predominantly expressed in the brain, but shorter one was dominant in the testis.

**Fig. 3:**
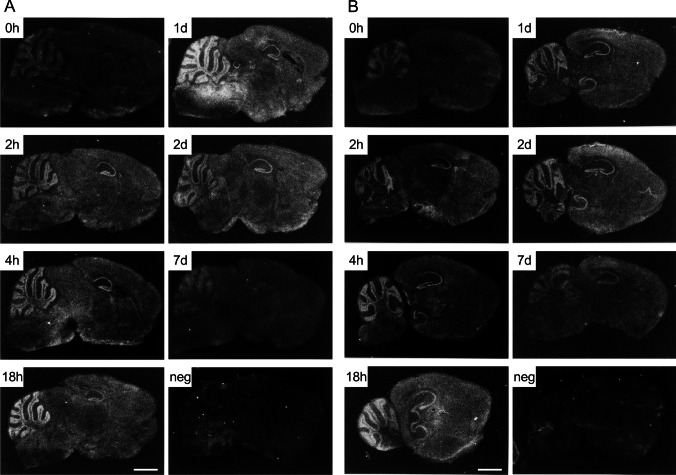
In situ hybridization histochemistry of differentially expressed 8CB + 8CC gene fragments, which later identified to Ndfip1 (N4WBP5) mRNA, at 2, 4, 18 h, 1, 2 or 7 days after single METH administration (20 mg/kg, i.p.) or saline administration (0 h). (*n* = 4 mice/each time point). **A**: medial sagittal sections (1.2 mm lateral to the midline) of mouse brain. **B**: lateral sagittal sections (2.3 mm lateral to the midline). Neg: hybridization with negative control sense probes. Scale bar = 2 mm

Figure 3 shows distribution of 8CB + 8CC mRNA, which later identified to Ndfip1, in the mouse sagittal brain slices of in situ hybridization histochemistry after single METH administration (20 mg/kg, i.p.). The mRNA rapidly induced in the hippocampus and cerebellum 2 h-2 days after the METH injection, peaked at 18 h, and gradually decreased to the control level at 7 days after injection of the drug. In the striatum and cerebral cortex, it can be seen at 18 h-2 days after METH administration, but was weaker than that in the hippocampus and the cerebellum.

### Involvement of Ndfip1 in METH-induced Neurotoxicity in Cultured Neuronal Cells

**Fig. 4:**
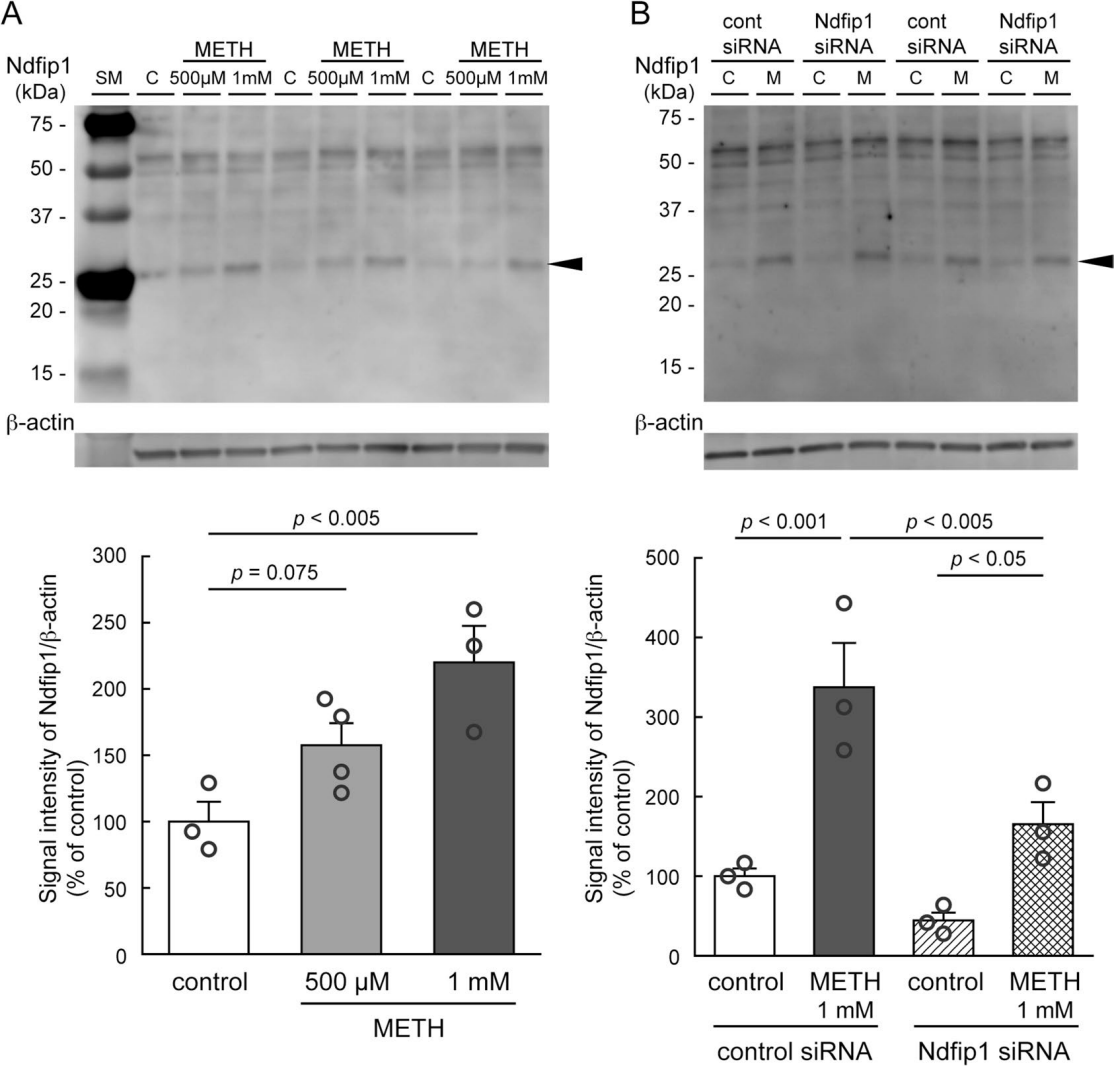
Western blot analyses of Ndfip1 (N4WBP5) protein in cultured neuronal cells after METH exposure and effects of Ndfip1 knockdown. **A**: Changes in Ndfip1 (N4WBP5) of B65 cells with METH treatment (500 µM or 1 mM) for 3 h. Each value is mean ± SEM (*n* = 3 ~ 4; *F*_(2,7)_ = 8.222, *p* = 0.0145; one-way ANOVA followed by Fisher’s PLSD test). **B**: Effects of Ndfip1 siRNA lipofection on Ndfip1 protein expression in B65 cells with/without METH treatment (1 mM) for 3 h. C: control, M: METH (1 mM). Arrowhead indicates specific band of Ndfip1 protein (26 kDa) Each value shows mean ± SEM (*n* = 3/each group; siRNA, *F*_(1,8)_ = 13.226, *p* = 0.0066; METH, *F*_(1,8)_ = 32.599, *p* = 0.0004; siRNA × METH, *F*_(1,8)_ = 3.441, *p* = 0.1007; two-way ANOVA followed by Fisher’s PLSD *post hoc* test). *P*-value was indicated between the two groups. For detailed statistical information, see Supplemental dataset file

**Fig. 5:**
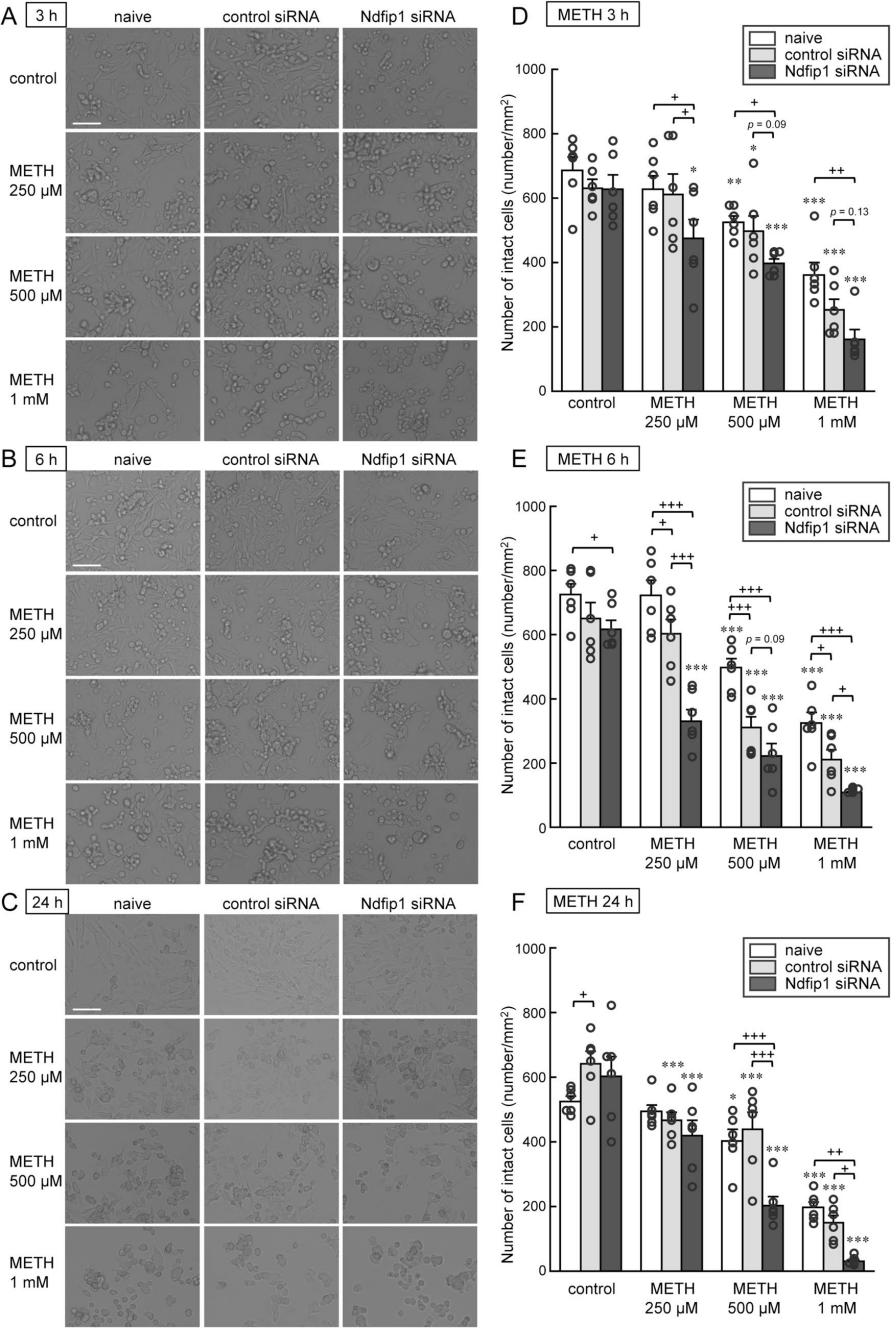
METH-induced neurotoxicity in monoaminergic B65 cells and effects of Ndfip1 (N4WBP5) knockdown. Representative photographs of B65 cells preincubated with negative control siRNA or Ndfip1 siRNAs after METH exposure (250, 500 µM or 1 mM) for 3 h (**A**), 6 h (**B**) or 24 h (**C**). Effects of lipofection with negative control siRNA or Ndfip1 siRNAs on the number of intact cells after METH exposure for 3 h (**D**), 6 h (**E**) or 24 h (**F**). Scale bar = 100 μm. Each value shows mean ± SEM (*n* = 6). Data were statistically analyzed by two-way ANOVA followed by Fisher’s PLSD *post hoc* test. At 3 h (**D**): siRNA, *F*_(2,60)_ = 10.897, *p* < 0.0001; METH, *F*_(3,60)_ = 50.469, *p* < 0.0001; siRNA × METH, *F*_(6,60)_ = 0.832, *p* = 0.5499. At 6 h (**E**): siRNA, *F*_(2,60)_ = 47.927, *p* < 0.0001; METH, *F*_(3,60)_ = 95.696, *p* < 0.0001; siRNA × METH, *F*_(6,60)_ = 3.429, *p* = 0.0056. At 24 h (**F**): siRNA, *F*_(2,60)_ = 11.515, *p* < 0.0001; METH, *F*_(3,60)_ = 96.443, *p* < 0.0001; siRNA × METH, *F*_(6,60)_ = 4.016, *p* = 0.0019. *p < 0.05, **p < 0.01, ***p < 0.001 vs. each control-treated group, #*p* < 0.05, ##*p* < 0.01, ###*p* < 0.001 between the two indicated groups. For detailed statistical information, see Supplemental dataset file

To investigate involvement of Ndfip1 (N4WBP5) expression in METH-induced neurotoxicity, we examined Ndfip1 expression in METH neurotoxicity and effects of knockdown of Ndfip1 expression with Ndfip1 siRNA using cultured monoaminergic neuronal B65 cells. Western blot analyses showed a specific band corresponding to Ndfip1 protein at expected size of 26 kDa (Fig. 4, arrowhead). The higher molecular weak bands at 34 kDa and 42 kDa reflect monoubiquitinated and diubiquitinated forms of Ndfip1 (N4WBP5). Upper bands at 50 kDa and 55 kDa are non-specific background (Harvey et al. 2002). METH exposure for 3 h dose-dependently increased Ndfip1 protein expression in the B65 cells (Fig. 4A: *F*_(2,7)_ = 8.222, *p* = 0.0145). The preincubation with Ndfip1 siRNAs significantly suppressed METH (1 mM)-induced Ndfip1 expression compared with that with negative control siRNA (Fig. 4B: siRNA, *F*_(1,8)_ = 13.226, *p* = 0.0066; METH, *F*_(1,8)_ = 32.599, *p* = 0.0004). The METH exposure for 3–24 h caused apparent morphological changes including cell loss, shrinkage of dendrites, shrunken round-shape cell body, or vacuolation in cytoplasm in the dose-dependent manner (Fig. 5A, B, and C). The number of intact B65 cells was dose-dependently decreased at 3 h, 6 h and 24 h after METH exposure (250, 500 µM or 1 mM) (Fig. 5D: METH, *F*_(3,60)_ = 50.469, *p* < 0.0001, Fig. 5E: METH, *F*_(3,60)_ = 95.696, *p* < 0.0001, and Fig. 5F: METH, *F*_(3,60)_ = 96.443, *p* < 0.0001). Furthermore, the METH-induced neurotoxicity at 6 h and 24 h was significantly aggravated in the B65 cells transfected with liposomed Ndfip1 siRNA compared with naïve cells or control siRNA-transfected cells (Fig. 5E: siRNA × METH, *F*_(6,60)_ = 3.429, *p* = 0.0056, and Fig. 5F: siRNA × METH, *F*_(6,60)_ = 4.016, *p* = 0.0019).

## Discussion

The main findings of this paper are that (1) DD RT-PCR method and sequent SSCP analysis revealed two differentially displayed cDNA fragments of ESTs in the mouse striatum after METH administration, which are later identified as mRNA of N4WBP5, later named Ndfip1; (2) drastic increases in Ndfip1 mRNA in the striatum after METH injections were confirmed; (3) a longer form of Ndfip1 mRNA was expressed in the brain; (4) the mRNA expression was increased in the hippocampus and cerebellum at 2 h-2 days, in the cerebral cortex and striatum at 18 h-2 days after single METH administration; and (5) METH exposure markedly increase Ndfip1 expression and the knockdown with Ndfip1 siRNA significantly aggravated METH-induced neurotoxicity in the cultured monoaminergic neuronal cells.

An E3 ubiquitin ligase Nedd4 is originally reported as a developmentally regulated gene highly expressed in embryonic brain (Kumar et al. 1992), but it is also expressed in various embryonic and adult tissues (Kumar et al. 1997). Nedd4 via its protein-protein interaction (WW) domains binds to the proline-rich PPxY (PY) motif of the target proteins for ubiquitination. One of the directly Nedd4-binding substrates is an epithelial sodium channel (ENaC). The WW domain of Nedd4 binds to PY motif of ENaC through ubiquitination to down-regulate ENaC activity (Abriel et al. 1999; Harvey et al. 2001). For the ubiquitination of many Nedd4 target proteins which have no PY motif, however, Nedd4 requires an adaptor protein Ndfip1 or Ndfip2 initially identified as Nedd4 WW domain-binding protein 5 (N4WBP5 or N4WBP5A), respectively (Harvey et al. 2002; Jolliffe et al. 2000; Shearwin-Whyatt et al. 2004). Ndfip1 (N4WBP5) contains two PY motifs, which the first PY motif highly interacts with the second and third WW domains of Nedd4, and three transmembrane regions, maintaining Golgi structure (Harvey et al. 2002), for the ubiquitination of Nedd4 substrate protein. Its mRNA ubiquitously expressed in most adult tissues such as brain, liver, kidney, heart, testis, prostate, salivary gland, pancreas and adult placenta in multiple tissue Northern blot analysis, with high expression in adult brain tissues, especially cerebellum > pituitary > thalamus > basal ganglia, cerebral cortex, hippocampus > striatum (Harvey et al. 2002). The analysis of mRNA expression pattern of our differentially displayed gene fragments, which later identified to Ndfip1 (N4WBP5), using Multiple Tissue Northern Blot (Fig. 2B) showed quite identical to the report by Harvey et al. (Harvey et al. 2002). Our in situ hybridization histochemistry shows similar localization pattern of the mRNA in the brain and its time-course changes. Ndfip1 mRNA is weakly but constitutively expressed in the cerebellum and cerebral cortex. The mRNA was remarkably induced in the hippocampus and cerebellum starting several hours after the single METH injection, and gradually increases in the striatum and cerebral cortex at 18 h-2 days (Fig. 3).

A number of studies showed involvement of dysfunction of UPS in METH-induced neurotoxicity. METH treatment induces formation of inclusion body, which contains ubiquitin, α-synuclein and parkin, and inhibits proteasome activity (Fornai et al. 2004a, b, 2005). METH-induced cell death was correlated with inhibition of proteasome (Lazzeri et al. 2006). Liao et al. reported that METH treatment decreased ubiquitin carboxy-terminal hydrolase L1 (UCH-L1), which heterozygous mutation causes autosomal dominant familial Parkinson’s disease Park 5, and increased level of α-synuclein in the striatum (Liao et al. 2005), suggesting that METH-induced downregulation of UCH-L1 impairs degradation of α-synuclein (Wu et al. 2021). METH increased ubiquitin-conjugating enzyme E2N in the striatum and hippocampus (Li et al. 2008). Parkin, a RING domain-containing E3 ubiquitin ligase which mutation causes autosomal recessive juvenile Parkinson’s disease Park2, is involved in METH-induced neurotoxicity. METH-induced oxidative stress decreased parkin and proteasome activities (Moszczynska and Yamamoto 2011). Overexpression of parkin in the nigrostriatal dopaminergic pathway protected METH-induced dopaminergic neurotoxicity in the rat striatum (Liu et al. 2013). Furthermore, METH exposure caused neurotoxicity, increases in polyubiquitin and α-synuclein, and decrease in parkin expression in cultured neuronal cells, while overexpression of parkin protected attenuated increases in α-synuclein and neurotoxicity, suggesting parkin is protective factor against METH-induced dysfunction of α-synuclein degradation (Meng et al. 2020). Jiao et al. reported that Synovial apoptosis inhibitor 1 (SYVN1), an endoplasmic reticulum-associated degradation (ERAD) E3 ubiquitin ligase, was associated with METH-induced degradation of GABA_A_α1 in the dorsal striatum (Jiao et al. 2017). Toll-like receptor 4-Pellino E3 ubiquitin ligase 1 (Peli1) axis and consequent receptor-interacting protein kinase 1 (RIPK1) is involved in the METH-mediated neuroinflammation, because knockdown of Peli1 significantly attenuated upregulation of proinflammatory cytokines induced by METH (Xu et al. 2023; Yang et al. 2020). These previous reports indicate that METH exposure cause dysfunction of UPS. In the present study, METH exposure induced Ndfip1 expression in vivo and cultured neurons (Figs. 2, 3, 4 and 5). Therefore, they would be compensatory reactions to ameliorate METH-induced dysfunction of UPS.

Nedd4 subfamily as HECT E3 ubiquitin ligases plays an important role in neurodevelopment and neurodegeneration (Haouari et al. 2022). Nedd4-mediated ubiquitination facilitates endosomal sequestration of internalized α-synuclein (Sugeno et al. 2014), and overexpression of Nedd4 protects α-synuclein accumulation and its neurotoxicity (Davies et al. 2014). Furthermore, Nedd4 interacts with and destabilizes PTEN (phosphatase and tensin homology deleted on chromosome 10), a dual specificity phosphatase involved in downregulating cellular survival, by catalyzing its polyubiquitination, consequently to reduce apoptosis (Wang et al. 2007), and to promote axonal branching (Drinjakovic et al. 2010). Genetic deletion of Ndfip1 revealed that Ndfip1 promotes PTEN ubiquitination and its nuclear trafficking (Li et al. 2014), to promote neuronal survival after cerebral ischemia (Howitt et al. 2012). Sang et al. found that Ndfip1 (N4WBP5) expression increased in the cerebral cortex after acute brain injury, and its overexpression increased the number of surviving neurons in vitro model of neuronal damage (Sang et al. 2006). Ischemia-induced Ndfip1 upregulated neuronal surviving by interacting with Nedd4-2 but not with other Nedd4 family Nedd-1 or Itch (Lackovic et al. 2012). A mitochondrial complex I inhibitor rotenone-induced neurotoxicity with reduction of Ndfip1 expression and increases in α-synuclein expression in cultured neuroblastoma cells were prevented by high expression of Ndfip1, suggesting that upregulation of Ndfip1 after rotenone exposure is adaptive or protective response to the toxicity (Liu et al. 2020). Foot et al. found that Ndfip1 and Ndfip2 (N4WBP5 and N4WBP5A) bind to divalent metal ion transporter DMT1, which transport non-heme iron into the cells, and act as adaptors between DMT1 and Nedd4 family E3 Nedd4-2 and WWP2, mediating ubiquitination and subsequent degradation of DMT1 to maintain iron homeostasis (Foot et al. 2008, 2011). In response to metal ion exposure to neuronal cells, Ndfip1 is upregulated and promotes ubiquitination of DMT1 by E3 Nedd4-2 to prevent metal toxicity (Howitt et al. 2009). Ndfip1 and iron concentration are increased in the substantia nigra of parkinsonian brain, the protein is expressed in dopaminergic neurons containing α-synuclein deposits and astrocytes (Howitt et al. 2014). Furthermore, Ndfip1 protects dopaminergic neuronal cells from iron-induced neurotoxicity by enhancing degradation of DMT1 (Howitt et al. 2014; Jia et al. 2015).

In the present study, METH treatment caused drastic and lasting increases in Ndfip1 mRNA in the striatum, and knockdown of Ndfip1 expression aggravated METH-induced neurotoxicity in the cultured neuronal cells. This is the first study to clarify the protective role of Ndfip1 against METH-induced neurotoxicity. Taken together with above mentioned previous reports showing cytoprotective profiles of Ndfip1, the marked increases in Ndfip1 expression after METH exposure in the present study might be compensatory reaction to protect neurons against METH-induced dysfunction of UPS and its neurotoxicity.

## Supplementary Information

Below is the link to the electronic supplemental dataset.

ESM 1 (PDF 69.8 KB)

## Data Availability

Results of all statistical analyses are provided in Supplemental dataset file. All other data generated during the current study are available from the corresponding author on reasonable request.
