# Supplementary material for: Differentially Expressed Nedd4-binding Protein Ndfip1 Protects Neurons Against Methamphetamine-induced Neurotoxicity
Source: Neurotox Res. 2025 Jan 14;43(1):4. doi: 10.1007/s12640-024-00725-z (PMC11732889; doi:10.1007/s12640-024-00725-z)
Supplement: Supplementary file 1 — (PDF 69.8 KB) [file 12640_2024_725_MOESM1_ESM.pdf]

Figure number		group	n	P value	F/t Values	Test type
1	DD analysis and SSCP					
		saline	5	mixed eaqual amount of striatal total RNA extracts (0.67 µg/µl)<br>from the striatum of 5 mice in each group		
		METH (5 mg/kg x4)	5			
2A	Northern blot hybridization					
		saline	5	10 µg of total RNA extracted from the striatum was blotted		
		METH (5 mg/kg x4)	5			
3	In situ hybridization histochemistry					
		saline	4			
		METH (20 mg/kg x1)	4			
4A	Western blot analyses of Ndfip1in B65 cells<br>Changes in Ndfip1 after METH treatment			p = 0.0145	F (2,7) = 8.222	one-way ANOVA followed by post-hoc Fisher's PLSD test
		control	3			
		METH (500 µM)	4	p = 0.075 (vs. control group)		
		METH (1 mM)	3	p = 0.0048 (vs. control group)<br>p = 0.0594 (vs. METH (500 µM) group)		
4B	Western blot analyses of Ndfip1in B65 cells<br>Effects of Ndfip1 siRNA on Ndfip1 with/without METH treatment			p = 0.0066 (siRNA)<br>p = 0.0004 (METH)<br>p = 0.1007 (siRNA x METH)	siRNA: F (1,8) = 13.226<br>METH: F (1,8) = 32.599<br>siRNA x METH: F (1,8) = 3.441	two-way ANOVA followed by post-hoc Fisher's PLSD test
	control siRNA	control	3			
		METH (1 mM)	3	p = 0.0007 (vs. control siRNA and control-treated group)		
	Ndfip1 siRNA	control	3	p = 0.2432 (vs. control siRNA and control-treated group)		
		METH (1 mM)	3	p = 0.0260 (vs. Ndfip1 siRNA and control-treated group)<br>p = 0.0047 (vs. control siRNAand METH-treated group)		
5D	Effects of Ndfip1 siRNA on the number of intact B65 cells<br>3 h after METH treatment			p < 0.0001 (siRNA)<br>p < 0.0001 (METH)<br>p = 0.5499 (siRNA x METH)	siRNA: F (2,60) = 10.897<br>METH: F (3,60) = 50.469<br>siRNA x METH: F (6,60) = 0.832	two-way ANOVA followed by post-hoc Fisher's PLSD test
	naïve	control	6			
		METH (250 µM)	6	p = 0.3269 (vs. naïve contro group)		
		METH (500 µM)	6	p = 0.0076 (vs. naïve control group)		
		METH (1 mM)	6	p < 0.0001 (vs. naïve control group)		
	control siRNA	control	6	p = 0.3585 (vs. naive control group)		
		METH (250 µM)	6	p = 0.7279 (vs. control siRNA control group)<br>p = 0.7755 (vs. naive METH (250 µM) group)		
		METH (500 µM)	6	p = 0.0237 (vs. control siRNA control group)<br>p = 0.632 (vs. naive METH (500 µM) group)		
		METH (1 mM)	6	p < 0.0001 (vs. control siRNA control group)<br>p = 0.0650 (vs. naive METH (1 mM) group)		
	Ndfip1 siRNA	control	6	p = 0.3283 (vs. naive control group)<br>p = 0.9522 (vs. control siRNA control group)		
		METH (250 µM)	6	p = 0.0103 (vs. Ndfip1 siRNA control group)<br>p = 0.0104 (vs. naive METH (250 µM) group)<br>p = 0.0215 (vs. control siRNA METH (250 µM) group)		
		METH (500 µM)	6	p = 0.0002 (vs. Ndfip1 siRNA control group)<br>p = 0.0323 (vs. naive METH (500 µM) group)<br>p = 0.0924 (vs. control siRNA METH (500 µM) group)		
		METH (1 mM)	6	p < 0.0001 (vs. Ndfip1 siRNA control group)<br>p = 0.0011 (vs. naive METH (1 mM) group)<br>p = 0.1257 (vs. control siRNA METH (1 mM) group)		
5E	Effects of Ndfip1 siRNA on the number of intact B65 cells<br>6 h after METH treatment			p < 0.0001 (siRNA)<br>p < 0.0001 (METH)<br>p = 0.0056 (siRNA x METH)	siRNA: F (2,60) = 47.927<br>METH: F (3,60) = 95.696<br>siRNA x METH: F (6,60) = 3.429	two-way ANOVA followed by post-hoc Fisher's PLSD test
	naïve	control	6			
		METH (250 µM)	6	p = 0.9712 (vs. naïve contro group)		
		METH (500 µM)	6	p < 0.0001 (vs. naïve control group)		
		METH (1 mM)	6	p < 0.0001 (vs. naïve control group)		
	control siRNA	control	6	p = 0.1439 (vs. naive control group)		
		METH (250 µM)	6	p = 0.3504 (vs. control siRNA control group)<br>p = 0.0202 (vs. naive METH (250 µM) group)		
		METH (500 µM)	6	p < 0.0001 (vs. control siRNA control group)<br>p = 0.0005 (vs. naive METH (500 µM) group)		
		METH (1 mM)	6	p < 0.0001 (vs. control siRNA control group)<br>p = 0.0308 (vs. naive METH (1 mM) group)		
	Ndfip1 siRNA	control	6	p = 0.0349 (vs. naive control group)<br>p = 0.5004 (vs. control siRNA control group)		
		METH (250 µM)	6	p < 0.0001 (vs. Ndfip1 siRNA control group)<br>p < 0.0001 (vs. naive METH (250 µM) group)<br>p < 0.0001 (vs. control siRNA METH (250 µM) group)		
		METH (500 µM)	6	p < 0.0001 (vs. Ndfip1 siRNA control group)<br>p < 0.0001 (vs. naive METH (500 µM) group)<br>p = 0.0910 (vs. control siRNA METH (500 µM) group)		
		METH (1 mM)	6	p < 0.0001 (vs. Ndfip1 siRNA control group)<br>p < 0.0001 (vs. naive METH (1 mM) group)<br>p = 0.0415 (vs. control siRNA METH (1 mM) group)		
5F	Effects of Ndfip1 siRNA on the number of intact B65 cells<br>24 h after METH treatment			p < 0.0001 (siRNA)<br>p < 0.0001 (METH)<br>p = 0.0019 (siRNA x METH)	siRNA: F (2,60) = 11.515<br>METH: F (3,60) = 96.443<br>siRNA x METH: F (6,60) = 4.016	two-way ANOVA followed by post-hoc Fisher's PLSD test
	naïve	control	6			
		METH (250 µM)	6	p = 0.5279 (vs. naïve contro group)		
		METH (500 µM)	6	p = 0.0159 (vs. naïve control group)		
		METH (1 mM)	6	p < 0.0001 (vs. naïve control group)		
	control siRNA	control	6	p = 0.0216 (vs. naive control group)		
		METH (250 µM)	6	p = 0.0008 (vs. control siRNA control group)<br>p = 0.5936 (vs. naive METH (250 µM) group)		
		METH (500 µM)	6	p = 0.0001 (vs. control siRNA control group)<br>p = 0.4786 (vs. naive METH (500 µM) group)		
		METH (1 mM)	6	p < 0.0001 (vs. control siRNA control group)<br>p = 0.3456 (vs. naive METH (1 mM) group)		
	Ndfip1 siRNA	control	6	p = 0.1166 (vs. naive control group)<br>p = 0.4459 (vs. control siRNA control group)		
		METH (250 µM)	6	p = 0.0004 (vs. Ndfip1 siRNA control group)<br>p = 0.131 (vs. naive METH (250 µM) group)<br>p = 0.3238 (vs. control siRNA METH (250 µM) group)		
		METH (500 µM)	6	p < 0.0001 (vs. Ndfip1 siRNA control group)<br>p = 0.0001 (vs. naive METH (500 µM) group)<br>p < 0.0001 (vs. control siRNA METH (500 µM) group)		
		METH (1 mM)	6	p < 0.0001 (vs. Ndfip1 siRNA control group)<br>p = 0.0012 (vs. naive METH (1 mM) group)<br>p = 0.0175 (vs. control siRNA METH (1 mM) group)		
